# Yeast strain development and process intensification in high-gravity fermentation

**DOI:** 10.1093/femsyr/foag046

**Published:** 2026-09-11

**Authors:** Son Chu-Ky, Tuan-Anh Pham, Ngoc-Hung Pham, Tien-Thanh Nguyen, Chinh-Nghia Nguyen, Tien-Cuong Nguyen, Tuan Le, Thuy-Hang Dam, Lan-Huong Nguyen, Thi Minh Tu Nguyen, Thi Anh-Tuyet Nguyen, Lan-Huong Phung, Quyet-Tien Phi, Quynh-Anh Pham, Quoc-Phong Truong, Kien-Cuong Pham, Luong Hung Tien, Minh Nhat Dang, Tien-Nam Tien, Joe Eun Son, Sung Keun Jung, Ui Wook Hwang, Soo Rin Kim

**Affiliations:** School of Chemistry and Life Sciences (SCLS), Hanoi University of Science and Technology (HUST), Hanoi 100000, Vietnam; Laboratory of Life Sciences and Genetic Resources Conservation, Hanoi University of Science and Technology (HUST), Hanoi 100000, Vietnam; School of Chemistry and Life Sciences (SCLS), Hanoi University of Science and Technology (HUST), Hanoi 100000, Vietnam; Laboratory of Life Sciences and Genetic Resources Conservation, Hanoi University of Science and Technology (HUST), Hanoi 100000, Vietnam; School of Chemistry and Life Sciences (SCLS), Hanoi University of Science and Technology (HUST), Hanoi 100000, Vietnam; Laboratory of Life Sciences and Genetic Resources Conservation, Hanoi University of Science and Technology (HUST), Hanoi 100000, Vietnam; School of Chemistry and Life Sciences (SCLS), Hanoi University of Science and Technology (HUST), Hanoi 100000, Vietnam; Laboratory of Life Sciences and Genetic Resources Conservation, Hanoi University of Science and Technology (HUST), Hanoi 100000, Vietnam; School of Chemistry and Life Sciences (SCLS), Hanoi University of Science and Technology (HUST), Hanoi 100000, Vietnam; Laboratory of Life Sciences and Genetic Resources Conservation, Hanoi University of Science and Technology (HUST), Hanoi 100000, Vietnam; School of Chemistry and Life Sciences (SCLS), Hanoi University of Science and Technology (HUST), Hanoi 100000, Vietnam; Laboratory of Life Sciences and Genetic Resources Conservation, Hanoi University of Science and Technology (HUST), Hanoi 100000, Vietnam; School of Chemistry and Life Sciences (SCLS), Hanoi University of Science and Technology (HUST), Hanoi 100000, Vietnam; Laboratory of Life Sciences and Genetic Resources Conservation, Hanoi University of Science and Technology (HUST), Hanoi 100000, Vietnam; School of Chemistry and Life Sciences (SCLS), Hanoi University of Science and Technology (HUST), Hanoi 100000, Vietnam; Laboratory of Life Sciences and Genetic Resources Conservation, Hanoi University of Science and Technology (HUST), Hanoi 100000, Vietnam; School of Chemistry and Life Sciences (SCLS), Hanoi University of Science and Technology (HUST), Hanoi 100000, Vietnam; Laboratory of Life Sciences and Genetic Resources Conservation, Hanoi University of Science and Technology (HUST), Hanoi 100000, Vietnam; School of Chemistry and Life Sciences (SCLS), Hanoi University of Science and Technology (HUST), Hanoi 100000, Vietnam; School of Chemistry and Life Sciences (SCLS), Hanoi University of Science and Technology (HUST), Hanoi 100000, Vietnam; School of Chemistry and Life Sciences (SCLS), Hanoi University of Science and Technology (HUST), Hanoi 100000, Vietnam; Institute of Biology, Vietnam Academy of Science and Technology (VAST), Hanoi 100000, Vietnam; Institute of Biology, Vietnam Academy of Science and Technology (VAST), Hanoi 100000, Vietnam; School of Chemistry and Life Sciences (SCLS), Hanoi University of Science and Technology (HUST), Hanoi 100000, Vietnam; Laboratory of Life Sciences and Genetic Resources Conservation, Hanoi University of Science and Technology (HUST), Hanoi 100000, Vietnam; Institute of Health Science and Technology, Hanoi University of Science and Technology (HUST), Hanoi 100000, Vietnam; Institute of Materials, Biology and Environment, Academy of Military Science and Technology, Hanoi 100000, Vietnam; Institute of Biotechnology and Food Technology, Thai Nguyen University, Thai Nguyen 250000, Vietnam; Faculty of Chemical Engineering, University of Science and Technology, The University of Da Nang, Da Nang550000,Vietnam; Center for Experiment and Practice, Ho Chi Minh City University of Industry and Trade (HUIT), Ho Chi Minh 700000, Vietnam; Department of Advanced Bioconvergence, Kyungpook National University, Daegu 41566, Republic of Korea; Department of Advanced Bioconvergence, Kyungpook National University, Daegu 41566, Republic of Korea; Department of Advanced Bioconvergence, Kyungpook National University, Daegu 41566, Republic of Korea; Department of Advanced Bioconvergence, Kyungpook National University, Daegu 41566, Republic of Korea

**Keywords:** high-gravity fermentation, very-high-gravity fermentation, yeast strain development, food and beverage bioprocessing, circular bioeconomy

## Abstract

High- and very-high-gravity (HG/VHG) fermentation increases substrate loading and product titers, thereby improving fermenter utilization and potentially reducing water use and downstream processing requirements. Initially developed for brewing and fuel ethanol production, these approaches are now applied more broadly in food, beverage, and bioproduct manufacturing. This MiniReview summarises operational definitions and industrial drivers of HG/VHG fermentation and examines the associated constraints in rheology, mass and heat transfer, osmotic and ethanol stress, nutrient availability, and oxidative damage. Yeast improvement strategies are reviewed, including adaptive laboratory evolution, mutagenesis, genome shuffling, multiplex genome editing, non-conventional yeasts, and multi-omics-guided selection. Process developments such as no-cook simultaneous liquefaction, saccharification and fermentation, enzyme formulation, nutrient management, and in situ product recovery are considered together with applications in alcoholic beverages, organic acids, microbial lipids, and other value-added products. The review also discusses coproduct valorization and the need to integrate strain development with process design. Current evidence supports HG/VHG fermentation as a useful process-intensification platform, although performance and sustainability depend strongly on feedstock, operating conditions, product requirements, and the basis used to report fermentation outcomes.

## Teaser

This MiniReview explains how improved yeasts and processing can make concentrated food and beverage fermentations more productive while limiting cellular stress, quality loss, and resource use.

## Introduction

In industrial fermentation, gravity denotes the concentration of dissolved solids, primarily fermentable sugars, and is commonly expressed as specific gravity or degrees Plato (°P). Definitions vary by sector: brewing generally considers 13–18°P to be high gravity (HG), whereas ethanol processes commonly use ∼150–250 g l⁻¹ fermentable sugar; very-high-gravity (VHG) fermentation typically exceeds 250 g l⁻¹ and can produce more than 15% (v/v) ethanol, although the precise threshold depends on the feedstock and application (Puligundla et al. 2011, Puligundla et al. 2019). Representative operating ranges are summarized in Table 1. HG fermentation was adopted in brewing to increase fermenter throughput by producing concentrated beer for subsequent dilution (Puligundla et al. 2011), and fuel ethanol producers similarly increased substrate loading in the expectation of expanding output without proportional investment in fermentation capacity and of reducing the energy required to recover ethanol from dilute broths.

**Table 1:** Operational ranges and representative examples of high-gravity (HG) and very-high-gravity (VHG) ethanol fermentation.

Category	Substrate	Fermentable sugar loading (g l⁻¹)	Ethanol (g l⁻¹)	Fermentation efficiency (%)^d^	Reference
HG	–	150–250	<118^c^	–	–
HG examples	Glucose	201	93	90.7	(Liu et al. 2012)
	Sugarcane molasses	240^a^	101	82.6	(Pradeep and Reddy 2010)
VHG	–	>250	>118	–	–
VHG examples	Glucose	298	131	86.2	(Liu et al. 2012)
	Sugar beet syrup	255^b^	120	87.5	(Joannis-Cassan et al. 2014)

a Reported as 24% (w/v) total reducing sugars (TRS) in a 33–34 °Bx molasses medium.

b Total sugar supplied during fed-batch fermentation.

c An ethanol concentration of 15% (v/v) calculated using an ethanol density of 0.789 g ml⁻¹ at 20°C. This value is used for descriptive convenience and does not represent a physiological or operational threshold.

d Fermentation efficiency (%) = (actual ethanol concentration/theoretical maximum ethanol concentration) × 100. The theoretical maximum ethanol yields were taken as 0.511 g ethanol g⁻¹ fermentable hexose (glucose and fructose) and 0.538 g ethanol g⁻¹ sucrose.

The principal rationale for HG/VHG fermentation is therefore to increase volumetric process efficiency while reducing the quantities of water, effluent, and downstream process streams handled per unit of product. Reported estimates from starch- and wort-based processes indicate process-water savings on the order of 40%–60% and energy-cost savings of ∼4% in specific cases (Puligundla et al. 2011). These values should not be interpreted as universal performance benchmarks, because the net economic and environmental outcomes depend on conversion yield, enzyme and energy inputs, system boundaries, and the treatment of coproducts (Janssen et al. 2016). The benefits of HG/VHG fermentation should therefore be assessed against an appropriate conventional-process control.

The relevance of high-substrate fermentation extends beyond beer and fuel ethanol, although the associated benefits and constraints remain application-specific. In brewing, elevated gravity alters carbon flux, redox balance, and amino-acid metabolism, thereby affecting ester and higher-alcohol formation and requiring coordinated control of temperature, oxygen availability, pitching rate, and nutrients (Casey et al. 1984, Saerens et al. 2008, Pires et al. 2014, Kinčl et al. 2021). High-sugar grape musts and concentrated fruit juices likewise expose yeast to strong osmotic stress initially and high ethanol concentrations later, increasing the risk of sluggish or incomplete fermentation (Adamczyk et al. 2016). Related high-substrate strategies are also relevant to organic acid and microbial lipid production, where increased volumetric productivity must be balanced against oxygen-transfer limitations, product inhibition, and downstream-recovery requirements (Papagianni 2007, Louhasakul et al. 2020). Yeast development has accordingly progressed from empirical selection of robust *Saccharomyces cerevisiae* strains to approaches combining adaptive evolution, genome engineering, and multi-omics analysis, while non-conventional yeasts broaden the range of usable substrates and provide diverse stress-tolerance traits, flavour profiles, and product-synthesis capabilities (Puligundla et al. 2019).

**Figure 1:**
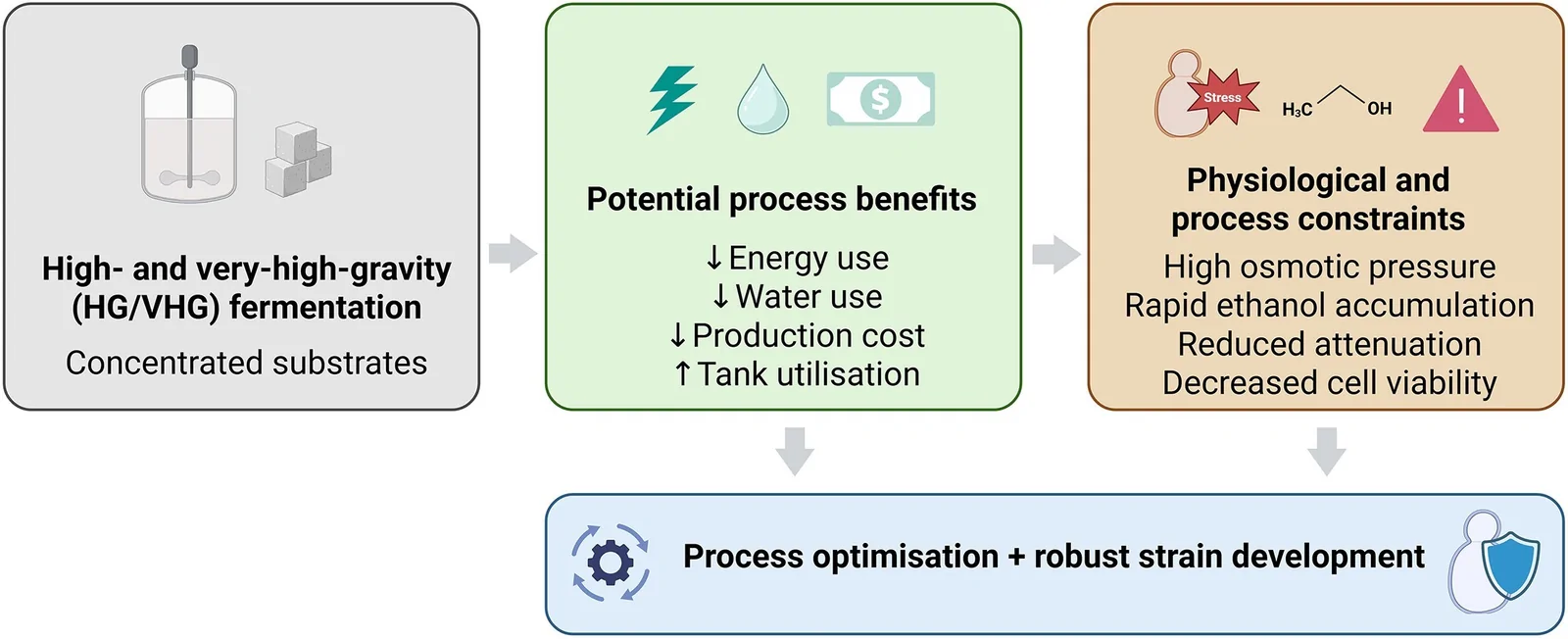
Potential process benefits and physiological constraints associated with high-gravity (HG) and very-high-gravity (VHG) fermentation. Created in BioRender. Kim, S. R. (2026) https://BioRender.com/rasr2ym.

This MiniReview synthesizes current understanding of HG/VHG fermentation across four interrelated dimensions: cellular physiology, strain development, process and enzyme engineering, and application-dependent performance. Particular emphasis is placed on the interactions between microbial robustness and process conditions, including substrate loading, nutrient availability, mass and heat transfer, product inhibition, and downstream requirements. Collectively, these perspectives provide a systems-level framework for evaluating the trade-offs that determine the performance, scalability, and sustainability of HG/VHG fermentation (Fig. 1).

## Physiological barriers and stress mechanisms in HG/VHG fermentation

### Rheology and mass transfer

High concentrations of substrate and suspended solids alter broth rheology and create heterogeneous conditions within the bioreactor. Above ∼30%–35% (w/w) solids, starch- and lignocellulose-containing media may exhibit high apparent viscosity, shear-thinning behaviour, and yield stress, depending on feedstock composition and particle structure (Mak 1992, Koppram et al. 2014). Poor circulation then promotes gradients in substrate, nutrients, dissolved gases, pH, temperature, and inhibitory metabolites (Hickman 1985, Bach et al. 2017), so cells experience different local environments within the same vessel, contributing to variable metabolic activity, incomplete sugar utilization, and sluggish or stuck fermentation (Olsvik and Kristiansen 1992, Huuskonen et al. 2010).

The severity of these limitations depends on the feedstock and pretreatment. Suspended starch, proteins, fibres, and insoluble particles can shift local flow from turbulent toward transitional or laminar conditions, while lignocellulosic fibre networks produce particularly pronounced non-Newtonian behaviour (Mak 1992, Koppram et al. 2014). Even at similar impeller speeds and aeration rates, high-viscosity media develop greater spatial heterogeneity than low-gravity broths (Hickman 1985, Bach et al. 2017). Suitable impeller and sparger configurations, staged feeding, controlled liquefaction, and feedstock-specific enzyme treatment can mitigate these effects, although benefits must be weighed against shear, foaming, and energy consumption. In no-cook simultaneous liquefaction, saccharification, and fermentation (SLSF), avoiding starch gelatinization limits formation of a highly viscous paste and facilitates mixing at high solids loading (Chu-Ky et al. 2016, Vu et al. 2023).

Although ethanol production is predominantly anaerobic, oxygen is required during propagation for sterol and unsaturated-fatty-acid synthesis, which contributes to subsequent ethanol tolerance (Aguilera et al. 2006, Puligundla et al. 2011). Insufficient oxygen can limit membrane-lipid synthesis and increase susceptibility to ethanol damage (Aguilera et al. 2006, Dong et al. 2015), while oxygen supply also affects redox balance and flavour-active metabolites in brewing (Saerens et al. 2008, Pires et al. 2014). Oxygen transfer becomes increasingly constrained as viscosity and suspended-solids loading rise, reducing the volumetric oxygen-transfer coefficient, *k*_L_*a*, which is the product of the liquid-phase mass-transfer coefficient, *k*_L_, and the gas–liquid interfacial area, *a* (Blanch and Bhavaraju 1976, Olsvik and Kristiansen 1992). Agitation and external circulation primarily increase *k*_L_, whereas improved sparger design, enhanced gas dispersion, and smaller bubbles increase *a*. Oxygen supplementation can further increase the transfer driving force, although these strategies must be balanced against energy demand, shear, and foaming.

Heat removal also becomes more difficult at high solids loading because fermentation is exothermic and elevated viscosity restricts convection and bulk mixing (Puligundla et al. 2011, Bach et al. 2017). Local overheating may increase membrane damage, oxidative stress, and ethanol toxicity even when the measured bulk temperature remains within the intended range; the risk is greater at large scale and where cooling-water temperatures approach the fermentation set point. Thermal management can combine appropriate temperature profiles, staged hydrolysis, internal or external heat exchange, and product recovery. Rheology, oxygen transfer, and heat dissipation should therefore be evaluated jointly during scale-up rather than extrapolated from low-solids laboratory media.

### Stressful environments

During VHG fermentation, yeast is exposed to high osmotic pressure at inoculation, progressively increasing ethanol concentrations during conversion, reduced water activity, nutrient imbalance, and oxidative damage; incomplete mixing can further intensify local exposure. Adaptation requires maintenance of turgor, membrane and ion homeostasis, protein stability, and redox balance through regulatory systems that include the high-osmolarity glycerol (HOG), cell wall integrity (CWI), protein kinase A, target of rapamycin, and general stress-response pathways (Hohmann 2002). High initial sugar concentrations cause water efflux, cell shrinkage, loss of turgor, and transient growth inhibition. In *S. cerevisiae*, the Sln1 and Sho1 sensing branches activate Pbs2 and Hog1, which coordinate transcription, cell-cycle control, and glycerol synthesis and retention (Davenport et al. 1999, Klipp et al. 2005). The magnitude and duration of HOG signalling depend on carbon source, metabolic state, and cell-to-cell variability (Babazadeh et al. 2017); glycerol supports osmoadaptation and redox balance but diverts carbon from ethanol, requiring a balance between protection and product yield (Puligundla et al. 2019).

As fermentation proceeds, ethanol becomes an increasingly important constraint because it accumulates continuously and alters both membrane structure and cellular energy demand. Ethanol partitions into the plasma membrane, disrupts sterol and phospholipid packing, and increases permeability (Cartwright et al. 1987). The resulting proton influx dissipates the electrochemical gradient and raises the ATP requirement for intracellular pH homeostasis, particularly at ethanol concentrations above ∼10% (v/v) (Rosa and Sá-Correia 1991, Madeira et al. 2010). Yeast responds by increasing plasma-membrane H⁺-ATPase activity and remodelling ergosterol and unsaturated fatty acids (Rosa and Sá-Correia 1991, Aguilera et al. 2006, Dong et al. 2015). High concentrations of dissolved sugars also reduce water activity and freely available water, affecting protein conformation, enzyme activity, membrane hydration, and growth (Hohmann 2002). Glycerol accumulation under osmotic stress is promoted by HOG-pathway activation and occurs mainly through Gpd1-dependent synthesis, whereas increased trehalose synthesis, including Tps1-dependent control, contributes to protection under ethanol stress; together, these compatible solutes support osmotic balance and stabilize proteins and membranes (Pallapati et al. 2021, Zhang et al. 2023).

The combined stress environment cannot be predicted reliably from assays of individual stressors because HOG and cell-wall signalling, membrane remodelling, antioxidant defence, and energy metabolism interact as conditions change (Rodríguez-Peña et al. 2010, de Nadal et al. 2011). Osmotic stress increases the requirement for compatible solutes, ethanol increases membrane permeability and ATP demand, and mitochondrial dysfunction promotes reactive oxygen species; these responses can reinforce one another during prolonged cultivation (Adamczyk et al. 2016, Charoenbhakdi et al. 2016). Screening and evolution conditions should therefore represent the intended HG/VHG process, and improved survival in a laboratory stress assay should be distinguished from improved sugar conversion, product titer, or yield.

Increasing carbon loading without a proportional increase in assimilable nutrients lowers the nutrient-to-carbon ratio and can restrict biomass formation and fermentation performance (Thomas and Ingledew 1990); nutrient supply is therefore treated as a process variable in Section 4.3.

Oxidative damage is particularly relevant when yeast is recycled through repeated fermentation cycles. Ethanol exposure, high metabolic activity, intermittent oxygen, and mitochondrial dysfunction promote reactive oxygen species and progressive loss of cellular fitness (Adamczyk et al. 2016, Liu et al. 2025). Yeast counteracts this damage through glutathione metabolism, superoxide dismutases, catalases, protein quality control, and autophagy (Adamczyk et al. 2016), but repeated use is associated with declining viability, an increased frequency of petite mutants, altered flocculation, and reduced fermentation performance (Huuskonen et al. 2010, Puligundla et al. 2011). Vacuolar H⁺-ATPase activity contributes to pH and ion homeostasis and can limit oxidative and cell-wall damage under ethanol stress (Charoenbhakdi et al. 2016). Evolutionary adaptation and targeted engineering may improve antioxidant capacity, but benefits should be evaluated across repeated cycles and against production metrics rather than survival alone (Adamczyk et al. 2016).

## Strategic yeast strain development: from classical to synthetic biology

### Non-GMO approaches: ALE, mutagenesis, and genome shuffling

Non-GMO approaches remain important in brewing, winemaking, and distilled-beverage production because they are generally compatible with existing regulations and consumer expectations. Adaptive laboratory evolution (ALE), random mutagenesis, and genome shuffling exploit natural or induced diversity without specifying individual genetic changes (Table 2). ALE enriches mutations that improve fitness under the imposed selection conditions; microdroplet ALE under ethanol stress improved fermentation of 28 °P wort, while chronic ethanol adaptation of *S. cerevisiae* CEN.PK 113–7D increased ethanol production from high-glucose medium (Yang et al. 2024, Sheikhi et al. 2025). In the CEN.PK 113–7D lineage, whole-genome sequencing identified mutations in the pentose phosphate pathway (*RKI1*), redox and mitochondrial function (*CYC2*), lipid metabolism (*LPX1*), and Rho-GTPase-dependent growth control (*RGA1, RGA2*), each of which improved growth at 9% (v/v) ethanol when reintroduced individually. However, the mutation conferring the highest ethanol tolerance differed from that producing the highest ethanol titer, indicating that these phenotypes were genetically separable. These results demonstrate the potential of ALE, although the selection environment should represent the combination and intensity of stresses encountered in the target process.

**Table 2:** Strain engineering strategies enhancing ethanol production during high-gravity (HG) and very-high-gravity (VHG) fermentation.

Engineering approach	Target or selection condition^a^	Parental strain	HG/VHG conditions^b^	Improved phenotype (compared to parent)^c^	Reference
Genome editing	Δ*gpd2*, Δ*fps1*, Δ*adh2*, Δ*dld3*, Δ*erg5*, Δ*nth1*, Δ*ams1*	*Saccharomyces cerevisiae* ATCC 204508	400 g l⁻¹ sucrose	135 g l⁻¹ ethanol (+17%)	(Yang et al. 2025)
			400 g l⁻¹ corn syrup (SSF)	145 g l⁻¹ ethanol (n.r.)	(Yang et al. 2025)
Random mutagenesis (ARTP)	Selection using a toxic proline analogue	*Saccharomyces pastorianus* YY (Industrial lager yeast)	33 °P	RDF 48.7% (+7.2%)	(Niu et al. 2025)
ALE (micro-droplet)	12% ethanol	*Saccharomyces cerevisiae* YN81 (Industrial brewing yeast)	28 °P	RDF 75.4% (n.r.); Tolerance to 12% ethanol (parent 10%)	(Yang et al. 2024)
ALE	11% ethanol	*Saccharomyces cerevisiae* CEN.PK 113–7D	220 g l⁻¹ glucose	94.5 g l⁻¹ ethanol (+20%)	(Sheikhi et al. 2025)
Genome shuffling	Ethanol productivity on xylose	*Saccharomyces cerevisiae* × *Scheffersomyces stipitis*	200 g l⁻¹ sugars (glucose:xylose = 3:1)	74 g l⁻¹ ethanol (+14%); Tolerance to 15% ethanol (parent 10%)	(Jetti et al. 2019)

Abbreviations: ARTP, Atmospheric and Room Temperature Plasma; ALE, adaptive laboratory evolution; SSF, simultaneous saccharification and fermentation; n.r., not reported.

a Ethanol concentrations used as selection conditions are expressed as % (v/v).

b P: Plato, g extract per 100 g of wort, mass percentage of dissolved solids (mainly fermentable sugars). Conversion to g l⁻¹ requires the wort specific gravity and is therefore not given.

c RDF is the real degree of fermentation (%), defined as the percentage of original extract consumed during fermentation; ethanol tolerance indicates the highest ethanol concentration at which growth was observed, and is given with the parental value for comparison.

Random mutagenesis similarly expands genetic diversity before phenotypic selection. Atmospheric and room-temperature plasma mutagenesis followed by selection with a toxic proline analogue produced *Saccharomyces pastorianus* variants with improved high-gravity brewing performance and increased intracellular proline, for which *GNP1* and *SUA7* were identified as the genes principally responsible (Niu et al. 2025). Proline functions as a compatible solute, so the selection enriched a trait relevant to the combined osmotic and ethanol stresses encountered during high-gravity brewing rather than to a single stressor. Genome shuffling combines favourable alleles from multiple parents through mating or protoplast fusion; hybridization of *S. cerevisiae* and *Scheffersomyces stipitis* improved fermentation of mixed glucose-xylose substrates at high sugar concentration (Jetti et al. 2019).

These methods provide broad phenotypic diversity but less genetic precision than targeted editing. High-throughput screening and systems-level analysis can improve their efficiency, while fermentation titer, yield, and product quality should be evaluated separately from growth or survival under isolated stress conditions.

### Rational and synthetic genome engineering

CRISPR-Cas systems enable precise and multiplex modification of loci involved in carbon partitioning, membrane composition, redox balance, and stress signalling, which is useful because HG/VHG performance is a quantitative trait determined by interacting pathways (Table 2). Simultaneous deletion of *GPD2, FPS1, ADH2, DLD3, ERG5, NTH1*, and *AMS1* in *S. cerevisiae* ATCC 204508 increased ethanol production from 400 g l⁻¹ sucrose and high-gravity corn syrup (Yang et al. 2025). The modifications reduced selected by-product pathways and altered sterol and reserve-carbohydrate metabolism. Additional targets include trehalose and glycerol metabolism, ergosterol and unsaturated-fatty-acid synthesis, antioxidant defence, and HOG-associated regulation (Hohmann 2002, Klipp et al. 2005); multiplex libraries can test combinations, but their value ultimately depends on performance under the relevant substrate, temperature, and product concentration.

Genome-scale rearrangement extends editing in a different direction. In Sc2.0 strains, Cre activation at loxPsym sites generates deletions, inversions, duplications, and other genome rearrangements. Screening of SCRaMbLEd populations yielded strains with increased tolerance to ethanol, heat, and acetic acid and identified *ACE2* as a negative regulator of ethanol tolerance; a loxPsym-mediated rearrangement removed its 3′ UTR and lowered expression (Luo et al. 2018). The resulting structural variants can be mapped by sequencing, although the synthetic chromosomes have primarily been studied in laboratory strain backgrounds.

Genome engineering can also modify product quality. In brewing, genes involved in higher-alcohol and ester formation, including *ARO10, BAT2, ATF1*, and *ATF2*, provide potential targets for controlling aroma under high-gravity conditions (Saerens et al. 2008, Pires et al. 2014). The use of genome-edited strains in foods and beverages remains dependent on regional regulation and consumer acceptance. Combining targeted engineering with ALE or phenotypic screening may improve industrial performance, but regulatory status and production benefits should be considered separately.

### Non-conventional yeasts in VHG applications

*Saccharomyces cerevisiae* remains the principal organism for alcoholic HG/VHG fermentation, but non-conventional yeasts provide complementary traits such as thermotolerance, broader substrate utilization, aroma formation, and synthesis of non-ethanol products. *Kluyveromyces marxianus* grows rapidly at elevated temperatures and utilizes substrates including lactose, inulin, and fructans, which can reduce cooling requirements and support simultaneous hydrolysis and fermentation of non-grain feedstocks; however, comparatively low ethanol tolerance remains a limitation for high-titer fermentation (Yuan et al. 2012). Ethanol tolerance increased from ∼7% to 10% (v/v) in strain FIM1 after ALE (Mo et al. 2019), whereas adaptation of MTCC1389 increased ethanol tolerance from 8% to 12% (v/v) and ethanol production by 42.9% (Pal and Vij 2022).

*Yarrowia lipolytica* is used primarily for lipids and organic acids rather than ethanol. Its capacity to redirect excess carbon under controlled nutrient limitation makes it relevant to high-solids bioprocessing and coproduct-oriented biorefineries (Louhasakul et al. 2020). Osmotolerant non-*Saccharomyces* yeasts, including *Meyerozyma caribbica*, may provide alternative alcoholic-fermentation hosts under high-sugar conditions (Matos et al. 2021), although their use requires control of population dynamics, nutrient demand, and fermentation completion.

Industrial adoption remains limited by less well-developed genetic tools, incomplete physiological characterization, and regulatory uncertainty. Selection of a non-conventional host should therefore be based on a defined process advantage rather than assumed general superiority to *S. cerevisiae*.

### From omics correlation to causative alleles

Robustness under HG/VHG conditions is generally polygenic, involving coordinated changes in stress signalling, membrane composition, redox balance, and energy metabolism (Klipp et al. 2005, Babazadeh et al. 2017). Multi-omics approaches can link these cellular responses to fermentation phenotypes and identify candidate traits for strain improvement. For example, combined genomic and transcriptomic analyses of evolved *K. marxianus* identified persistent changes in membrane-lipid synthesis and stress-response pathways, providing candidate functions for further strain engineering (Mo et al. 2019). Metabolic profiling of an independently evolved strain further associated ethanol tolerance with changes in ergosterol, fatty acids, and central metabolites (da Silveira et al. 2020). In engineered *S. cerevisiae*, targeted metabolomic analysis linked improved ethanol production to changes in glycerol, membrane-lipid, and reserve-carbohydrate metabolism (Yang et al. 2025). Together, these approaches can prioritize genes and pathways for subsequent engineering or evolutionary selection and provide mechanistic feedback for strain development.

QTL mapping by pooled-segregant whole-genome sequence analysis provides a route from polygenic phenotypes toward causal allele identification. Sequencing of segregant pools tolerant to ≥ 16% and ≥ 17% (v/v) ethanol resolved three major loci and additional minor loci and identified *MKT1, SWS2*, and *APJ1* within the locus of strongest linkage (Swinnen et al. 2012). The minor loci were more pronounced in the 17% pool, indicating that part of the genetic architecture becomes detectable only under stronger ethanol selection. Candidates can then be tested by reciprocal hemizygosity or allele replacement.

These approaches are complementary: genomic or QTL analysis identifies candidate genetic determinants, while transcriptomic and metabolomic analyses provide regulatory and physiological context for prioritization. Candidate validity should ultimately be confirmed by improved fermentation performance under process-relevant HG/VHG conditions.

## Innovations in processing and enzymatic systems

### No-cook simultaneous liquefaction, saccharification, and fermentation

Conventional starch-to-ethanol processing gelatinizes and liquefies starch at high temperature before saccharification and fermentation, requiring steam and subsequent cooling. No-cook SLSF instead uses raw-starch-hydrolysing enzymes that operate near fermentation temperature, allowing starch hydrolysis and fermentation to proceed in one vessel at ∼30°C–35°C (Xu and Duan 2010, Chu-Ky et al. 2016). Avoiding gelatinization limits the associated increase in mash viscosity and simplifies the heating and cooling sequence.

At 25-L pilot scale, 311.5 g l⁻¹ raw rice solids produced 17.0% (v/v) ethanol at 83.2% of the theoretical yield, compared with 17.6% (v/v) at 86.3% in the corresponding laboratory-scale fermentation (Chu-Ky et al. 2016). Subsequent optimization of enzyme dosage, protease supplementation, and inoculum size improved process performance and reduced raw-material cost (Vu et al. 2023). The resulting distillers' grains were protein-rich, although direct comparison with material from an otherwise equivalent cooked process is still required to attribute coproduct differences specifically to no-cook processing.

Raw-starch-hydrolysing enzymes and temperature profiling have also supported high-gravity fermentation of sorghum and other cereals, indicating that the approach is not restricted to rice (Xu and Duan 2010). Performance depends on starch structure, enzyme formulation, particle size, solids loading, and yeast strain. No-cook SLSF can therefore reduce thermal processing and facilitate high-solids starch fermentation, but its economic and environmental advantages should be quantified against conventional cooking using consistent boundaries that include enzyme use, fermentation performance, downstream recovery, and coproduct value.

### Advanced enzymatic cocktails: RSHE and proteases

Enzyme formulation is central to high-solids SLSF because it affects starch accessibility, sugar-release rate, and broth handling. Raw-starch-hydrolyzing enzyme (RSHE) preparations generally combine α-amylase and glucoamylase activities that act on native starch granules near fermentation temperature, avoiding prior gelatinization (Xu and Duan 2010). In rice-based VHG fermentation, commercial RSHE formulations supported ethanol concentrations above 17% (v/v), although performance depends on feedstock structure, particle size, solids loading, enzyme dose, and temperature (Vu et al. 2023).

Proteases complement starch-hydrolysing enzymes by releasing free amino nitrogen (FAN) and improving starch accessibility through degradation of the grain protein matrix. In rice VHG fermentation, protease supplementation increased FAN from 73 to 336 mg l⁻¹, raised the final ethanol concentration by 2.4% (v/v), and shortened fermentation by 48 h relative to the unsupplemented control, whereas urea had little effect (Tien et al. 2022). These effects likely reflect the combined benefits of increased nitrogen availability and improved starch accessibility. Other feedstocks require different accessory enzymes, including β-glucanases and xylanases in brewing and cellulase-hemicellulase systems for lignocellulosic materials. Enzyme cocktails should therefore be selected using combined criteria of conversion, viscosity, nutrient release, product quality, and cost rather than hydrolysis yield alone (Chatzifragkou et al. 2015).

### Nutrient limitation and fortification strategies

Increasing carbon loading without a proportional nutrient supply can limit biomass formation, sugar utilization, and stress tolerance. Nitrogen is commonly supplied as defined sources such as diammonium phosphate or ammonium sulfate, as complex sources such as corn steep liquor, yeast extract, or protein hydrolysates, or through enzymatic release from endogenous feedstock proteins. An optimized low-cost VHG medium containing corn steep liquor and urea together with MgSO₄·7H₂O and CuSO₄·5H₂O enabled the industrial strain PE-2 to produce 18.6% (v/v) ethanol from up to 330 g l⁻¹ glucose, at a reported ethanol yield of 93% (Pereira et al. 2010). Alternatively, protease-assisted release of endogenous nitrogen reduced reliance on external supplementation in rice SLSF and improved fermentation kinetics (Tien et al. 2022). Magnesium, zinc, and copper are generally supplied as soluble sulfate salts to meet mineral requirements, while oxygen is provided through controlled aeration during propagation or at the start of fermentation to support sterol and unsaturated-fatty-acid synthesis rather than by continuous aeration throughout ethanol production (Aguilera et al. 2006, Puligundla et al. 2019). Nutrient supplementation can improve fermentation performance in both high-gravity brewing and molasses systems (Casey et al. 1984, Pradeep and Reddy 2010); however, in brewing, nitrogen availability and oxygenation also influence attenuation and the formation of flavour-active metabolites (Saerens et al. 2008, Lei et al. 2012).

Nutrient requirements vary with feedstock, yeast strain, inoculum size, and fermentation stage, so fortification should be optimized for each process rather than applied as a fixed formulation. Because nutrient availability interacts with enzyme loading and inoculum size, these variables are often optimized jointly rather than independently. Design-of-experiments approaches can simultaneously optimize enzyme dose, nitrogen supply, inoculum, and operating conditions. In rice SLSF, a Taguchi L9 design coupled with a desirability function was used to optimize enzyme dosage and inoculum size, achieving 17.45 ± 0.07% (v/v) ethanol at 86.08 ± 0.20% fermentation efficiency and reducing raw-material cost by 7.6% (Vu et al. 2023). For food and beverage applications, the optimized regime should also meet sensory-quality and labelling requirements.

### Integrated in situ product recovery

As ethanol accumulates, membrane damage and increased cellular maintenance requirements can limit further conversion at high gravity. *In situ* product recovery (ISPR) addresses this limitation by removing ethanol during fermentation rather than after completion. The principal approaches are vacuum-assisted removal, membrane separation, and extraction; each can reduce product inhibition but introduces additional capital, energy, and operating requirements (Puligundla et al. 2011, Peng et al. 2021).

Vacuum-assisted fermentation promotes ethanol evaporation at reduced pressure and can extend productive fermentation, but pressure reduction also affects foaming, gas disengagement, heat transfer, and vapour handling (Puligundla et al. 2011). Its feasibility therefore depends on integrating vacuum generation, condensation, and heat recovery with reactor operation; these interactions are particularly important in viscous high-solids media (Campos et al. 2025).

Membrane-based separation, particularly pervaporation through hydrophobic membranes, removes ethanol without bulk boiling and retains cells and non-volatile nutrients (Peng et al. 2021). Composite membranes and immobilized-cell configurations have improved selectivity and productivity at laboratory scale (Cai et al. 2016). Industrial use nevertheless remains constrained by fouling, concentration polarization, cleaning requirements, and declining mass transfer in viscous broths (Peng et al. 2021).

Extractive fermentation transfers ethanol or other hydrophobic products to an immiscible phase. Performance depends on partition coefficient, solvent toxicity, recoverability, and cost; food and beverage applications additionally require food-compatible extraction media and control of sensory effects. ISPR should therefore be evaluated by overall fermentation and separation performance rather than product removal alone, including solvent or membrane recovery, energy integration, cleaning, and regulatory suitability.

## Industrial case studies and applications

### Rice-based potable ethanol: a pilot-scale VHG case study

Traditional rice-spirit production commonly combines cooking with relatively low-gravity fermentation and empirical process control. Rice-based VHG no-cook SLSF provides a representative example of translating the process strategies described in Section 4 into potable ethanol production at pilot scale (Chu-Ky et al. 2016, Tien et al. 2022, Vu et al. 2023). Together, raw-starch hydrolysis, nutrient release, and process optimization demonstrate how high solids loading can be implemented while maintaining fermentation performance. Although pilot-scale feasibility has been demonstrated, commercial adoption will depend on whether reduced thermal-processing requirements and improved fermentation performance offset enzyme costs while maintaining sensory quality and food safety, and therefore requires validation at commercial scale.

### High-gravity alcoholic beverages: brewing and wine

In brewing, concentrated wort can increase brewery throughput through post-fermentation dilution; however, increasing wort gravity from 14 to 24 °P reduced fermentability from 76% to 67% and increased ethyl acetate from 14.5 to 37.8–39.4 mg l⁻¹ when normalized to the same ethanol content (Piddocke et al. 2009).

A comparable limitation is observed in high-sugar wine fermentation. In Vidal ice-wine must, increasing the initial sugar concentration from 370 to 550 g l⁻¹ decreased ethanol production from 130.26 to 90.85 g l⁻¹ while increasing acetic acid from 0.82 to 2.86 g l⁻¹ (Zhang et al. 2024). At ≥500 g l⁻¹ sugar, fermentation was also associated with higher residual sugar and poorer sensory balance, illustrating that excessive substrate concentration can compromise both fermentation efficiency and product quality. Together, these studies indicate that the practical HG/VHG limit in alcoholic beverages reflects a balance between substrate loading, fermentation completion, and sensory quality.

### Bioethanol from agro-industrial waste

Agricultural and food-processing residues, including bagasse, straw, cassava residues, and oil-palm empty fruit bunches, provide potential substrates for high-solids ethanol production. Their conversion generally requires pretreatment and enzymatic hydrolysis, which increase sugar accessibility but may generate furans, weak acids, and phenolic inhibitors (Almeida et al. 2007, Jönsson and Martín 2016). At high solids loading, viscosity and heterogeneous mixing further limit enzyme and yeast performance, partly offsetting the benefits of higher product concentration (Puligundla et al. 2011).

This trade-off is illustrated by fed-batch SSF of alkali-pretreated sugarcane bagasse. At 30% (w/v) solids, 66.9 g l⁻¹ ethanol was produced at 72.9% conversion efficiency after 96 h, whereas increasing cumulative solids loading to 36% was accompanied by declining conversion efficiency (Liu et al. 2016). Thus, increasing solids loading beyond an optimal range may compromise conversion despite the potential for higher product concentration. Contamination control also remains important because lignocellulosic hydrolysate composition can differentially affect yeast and bacterial contaminants (Giacon et al. 2025). Industrial feasibility therefore depends on balancing ethanol concentration against conversion efficiency, inhibitor effects, and process operability.

### Extension beyond ethanol: organic acids and microbial lipids

High-substrate cultivation is also relevant to non-ethanol products in yeasts, although these processes are not conventionally classified as HG/VHG fermentation. The oleaginous yeast *Yarrowia lipolytica* provides examples for both organic acid and lipid production. At a total glycerol loading of 300 g l⁻¹, citric acid titers reached 155.2–157.5 g l⁻¹, whereas volumetric productivity was higher at 200 g l⁻¹ glycerol (0.94–1.05 g l⁻¹ h⁻¹) (Rywińska et al. 2010). Thus, increasing carbon loading improved product titer but diminished process productivity. A similar limitation is evident in microbial lipid production: high crude-glycerol concentrations progressively impaired growth of the parental *Y. lipolytica*, whereas an ALE-derived strain cultivated with 20% (v/v; ∼250 g l⁻¹) crude glycerol achieved approximately twofold higher biomass and lipid yields than the parental strain in a 3-L bioreactor (Tsirigka et al. 2023). These examples show that the practical substrate-loading limit depends on strain tolerance and product-specific cultivation conditions rather than substrate concentration alone.

### Scale-up, process control, and quality management

At production scale, HG/VHG fermentation requires operating conditions that maintain efficient substrate conversion without increasing undesirable by-products. In VHG fermentation of sugarcane molasses at 40 °Bx, aeration at 0.2 vvm produced 12.2% (v/v) ethanol and reduced residual sugar from 60.4 to 18.7 g l⁻¹ compared with fermentation without aeration, whereas further increases in aeration did not improve ethanol production and promoted by-product formation (Arshad et al. 2017). This illustrates that process variables should be maintained within an appropriate operating window rather than simply maximized.

For food and beverage applications, scale-up must also preserve product quality. In industrial high-gravity brewing with 15.5 °P wort in 3850-hL tanks, response-surface optimization of pitching rate, fermentation temperature, wort aeration, and tank-filling time identified 10 × 10⁶ cells ml⁻¹, 11.5°C, 12 mg l⁻¹ dissolved oxygen, and a 4.5-h filling time as conditions that minimized acetaldehyde and dimethyl sulfide while maximizing predicted sensory quality (Kucharczyk et al. 2020). Thus, production-scale control should integrate fermentation performance with monitoring of key quality and safety parameters. Hygienic design and HACCP- and GMP-based controls remain necessary because osmophilic or ethanol-tolerant spoilage organisms may persist under HG/VHG conditions (Puligundla et al. 2019).

## Sustainability and circular economy

### Coproduct valorization

The sustainability of HG/VHG fermentation depends not only on ethanol productivity but also on the utilization of fermentation coproducts. Distillers' dried grains (DDG) and distillers' dried grains with solubles (DDGS), traditionally used as animal feed, contain substantial protein, fibre, lipids, and minerals that can support higher-value applications (Chatzifragkou et al. 2015, Böttger and Südekum 2018). In rice-based SLSF-VHG fermentation, alkaline extraction increased DDG protein purity to ∼87% (dry basis), and incorporation of the recovered protein into bakery products did not adversely affect colour, texture, or sensory acceptance (Nguyen et al. 2021). This provides a direct example of converting an HG/VHG coproduct into a potential food ingredient.

Oleaginous yeasts may also be used to valorize carbon-rich residues from ethanol processes. For example, *Trichosporon cutaneum* utilized cellulosic ethanol fermentation stillage without pretreatment or external nutrient supplementation, producing 2.16 g l⁻¹ microbial lipid while reducing COD by 55.05% after 5 days in a 3-L bioreactor (Wang et al. 2017). This provides a complementary route for extending ethanol fermentation toward sequential carbon utilization, although the low lipid titer and laboratory scale indicate that further process development is required.

However, coproduct valorization does not inherently improve overall sustainability. Additional fractionation and purification require energy and chemicals, and their benefits depend on recovery yield, product value, and allocation of environmental burdens among ethanol and coproducts. Coproduct recovery should therefore be evaluated together with the primary fermentation process rather than assumed to provide an independent sustainability benefit (DeRose et al. 2019).

### Life-cycle implications of HG/VHG fermentation

Higher ethanol concentrations can reduce the amount of water requiring downstream removal, but these process-level advantages do not necessarily translate into lower life-cycle impacts. In an LCA of high-gravity ethanol production from spruce wood, a 30% dry-matter configuration reduced the amount of water requiring downstream removal from ∼25 to 10 L l⁻¹ ethanol relative to the low-solids base case. Nevertheless, only six of 27 evaluated configurations achieved a lower global warming potential (GWP), because environmental performance was strongly dependent on ethanol yield and process inputs (Janssen et al. 2016).

Enzyme use was particularly important, accounting for 67%–77% of GWP across the evaluated configurations, whereas on-site enzyme production substantially reduced this contribution (Janssen et al. 2016). An earlier assessment of wheat-straw ethanol similarly showed that increasing dry-matter content did not necessarily improve environmental performance when enzyme-related and other process burdens increased (Janssen et al. 2014). Thus, HG/VHG fermentation should not be considered intrinsically more sustainable than conventional fermentation; its environmental advantage depends on maintaining conversion yield while reducing water, energy, enzyme, and other material inputs, together with effective coproduct utilization. Feedstock- and region-specific assessments are therefore particularly needed for rice-, cassava-, and molasses-based HG/VHG processes.

## Conclusion and future prospects

HG/VHG fermentation can increase product concentration and fermenter utilization, but these benefits are accompanied by intensified osmotic and ethanol stress, nutrient limitation, and mass- and heat-transfer constraints at high substrate and solids loadings. The evidence reviewed here indicates that successful intensification requires coordinated optimization of strain robustness, substrate conversion, nutrient supply, process operation, and product quality rather than maximization of substrate concentration alone. The appropriate strategy is application-dependent: alcoholic beverages require preservation of fermentation completion and sensory quality, starch-based processes depend strongly on enzyme and nutrient management, and lignocellulosic systems must additionally address inhibitors and high-solids processing.

Future strain development will increasingly integrate evolutionary selection, genome editing, QTL mapping, multi-omics analyses, and Design-Build-Test-Learn workflows. AI and machine-learning approaches may accelerate this cycle by prioritizing genetic and process variables, but their predictive value will depend on sufficiently large, standardized datasets generated under industrially relevant HG/VHG conditions (Liao et al. 2022). Computational predictions should therefore complement, rather than replace, experimental validation of titer, yield, productivity, robustness, and product quality. Omics-derived candidates should likewise be causally validated and confirmed under process-relevant fermentation conditions.

At the process level, future progress should focus on integrating robust strains with well-controlled high-substrate fermentation, efficient downstream processing, and coproduct utilization. As demonstrated by the life-cycle evidence discussed in Section 6, higher gravity does not inherently confer environmental superiority; the benefit depends on maintaining conversion efficiency while controlling enzyme, energy, water, and material inputs. Future HG/VHG development should therefore be evaluated using application-specific technical, economic, quality, and environmental criteria. The long-term value of HG/VHG fermentation lies not simply in achieving higher substrate or product concentrations, but in delivering reproducible process intensification with demonstrable advantages at production scale.
